# Health-related quality of life and its influencing factors in patients with breast cancer based on the scale QLICP-BR

**DOI:** 10.1038/s41598-023-41809-8

**Published:** 2023-09-13

**Authors:** Shu Chen, Yuxi Liu, Daniel Yee Tak Fong, Jiali Zhou, Huanwei Chen, Chonghua Wan

**Affiliations:** 1 The First Dongguan Affiliated Hospital of Guangdong Medical University, Dongguan, China; 2 Key Laboratory for Quality of Life and Psychological Assessment and Intervention, Research Center for Quality of Life and Applied Psychology, Guangdong Medical University, Dongguan, China; 3 School of Nursing, The University of Hong Kong, Hong Kong, China; 4 Central Hospital of Guangdong Nongken, Zhanjiang, China

**Keywords:** Breast cancer, Oncology, Risk factors

## Abstract

Breast cancer is the most common cancer and the leading cause of cancer death among females worldwide. During the past 15 years, quality of life (QOL) has become an important aspect of breast cancer treatment. The purpose of this study was to evaluate QOL of breast cancer patients in China, and investigate its associations with sociodemographic and clinical variables. A cross-sectional study was conducted in 246 breast cancer patients in China. Recruited patients were surveyed for QOL using the QOL instruments for cancer patients-breast cancer QLICP-BR (V2.0). We assessed the associations between potential influencing factors and QOL using multiple linear regression models. The general mean QOL score for our population was 70.24 with SD = 8.70. Results indicated that medical insurance, drinking history, alkaline phosphatase, serum chloride ion level, serum calcium ion level, serum phosphorus ion level, mean corpuscular volume, mean corpuscular hemoglobin, red cell volume distribution width and platelet had significant associations with QOL of breast cancer patients. Our results emphasized that many factors are affecting QOL of breast cancer patients, which may provide a reference for targeted management or intervention strategies of breast cancer patients to improve their QOL.

## Introduction

Breast cancer is the most common cancer and the leading cause of cancer death among women worldwide^1^. In 2017, there were 1,960,682 new cases of breast cancer and 611,625 deaths globally. Moreover, over the past 30 years, new cases of breast cancer, and deaths have also been increased around the world^2^. Despite the incidence of breast cancer in China is lower than that in the western countries^3^, it has been increased much faster than that in the other countries. This may be due to the changes in diet, lifestyle, environment, and the unique one-child policy in China^4^. In 2017, there were 357,569 new female breast cancer patients in China, accounting for 19.1% of the total incidence of all types of cancer in women. Hence, breast cancer still present as a major public health issue^5^.

Continual technical advances in diagnosis technology and refinements in cancer treatment modalities have prolonged survival, and thus led to increasing number of breast cancer survivors^6^. In addition to survival, quality of life (QOL) has also been an important treatment target for breast cancer ^7,8^. The literature has well demonstrated that breast cancer may have substantial adverse influence on breast cancer patients' QOL, which encompasses physical, mental, emotional, and social functioning^9,10^.

In China, most studies have adopted translated versions of QOL instruments originally developed from the West^11,12^. For instances, the Chinese versions of the QLQ-C30, QLQ-BR23, and FACT-G and FACT-B have been widely used for assessing the QOL of Chinese breast cancer patients^11–14^. However, it has been highly recognized that there are substantial cultural differences between China and the West. For example, the family relationship and kinship play very important roles in daily life; Taoism and traditional medicine focus on good temper and high spirit; Good appetite, sleep, and energy are highly regarded in daily life with food culture being very important ^15,16^. The World Health Organization Quality of Life Assessment (WHOQOL) defines the quality of life as individual's perceptions of their position in life in the context of the culture and value systems in which they live and in relation to their goals, expectations, standards, and concerns^17^. The definition shows a high cultural dependence of QOL, and the understanding and interpretation of QOL should be deeply rooted in the local cultural background. Therefore, it is essential to develop a Chinese-specific QOL instrument system for cancer patients.

To address this need, a system of Chinese QOL instruments, namely the Quality of Life Instruments for Cancer Patients (QLICP), was developed by a modular approach^18,19^. In 2013, the first version of the QOL instruments system QLICP (V1.0) was developed, which was later revised as the second version of the QLICP (V2.0)^19^. The QLICP V2.0 includes the general scale (module) QLICP-GM that can be used with all types of cancer, and 22 cancer-specific scales, such as breast cancer (QLICP-BR), brain cancer (QLICP-BN), bladder cancer (QLICP-BL), prostate cancer (QLICP-PR), cervical cancer (QLICP-CE), leukemia (QLICP-LE) and lymphoma (QLICP-LY) ^19^. To date, most scales of the QLICP V2.0 have been developed and put into use^19^.

The QLICP-BR (V2.0) has distinct Chinese cultural characteristics like the QLICP-BR (V1.0)^15^. For example, the Chinese culture pays more attention to the family relationship and kinship, eating and food, good temper and high spirit, and it includes some items focusing on these such as appetite, sleep, energy, family support etc^15,16^. Therefore, it has been used extensively in China and has produced great social benefits^19–21^. However, there are no reports on the influencing factors of QOL based on the QLICP-BR (V2.0) measuring QOL in Chinese breast cancer patients to date.

This study aimed to describe the quality of life of Chinese breast cancer patients using QLICP-BR (V2.0), to investigate its association with socio-demographic and clinical variables, and to provide a reference for further research on the quality of life of breast cancer patients.

## Patients and methods

### Sample and setting

This current study is a cross-sectional study, which was approved by the Ethics Committees at all participating hospitals.

Study subjects were recruited at the Guangdong Nongken Hospital, the Affiliated Hospital of Guangdong Medical University, and the Yunnan Cancer Hospital from April 2019 to July 2020. The subjects in this study were breast cancer patients at any stage who were treated at above hospitals, and able to read and understand the scale during the survey period.

The inclusion criteria were: (1) Patients with a clear diagnosis as breast cancer by pathological examination; (2) Good reading and presentation skills, be able to fill out questionnaires by themselves; (3) Volunteer to participate in the survey, no mental illness or disturbance of consciousness. Exclusion criteria were having severe psychosomatic disease complications, illiteracy, or indication of non-cooperation with investigation.

The investigator explained the survey and scale to them and informed consent was obtained from the subjects. These participants were asked to complete the General Information Questionnaire and the QLICD-BR V2.0 during their interview with the investigator. The investigator immediately checked the answers each time to ensure completeness. If missing values were found, the questionnaire was returned to the patient to complete the missing items.

Depending on the empirical methodology, the sample size required for a multivariate analysis is usually 5–10 times the number of independent variables in the survey. Also some studies have recommended that any regression analysis should have a minimum of 200 study subjects^22^. In this study, considering maximum 48 independent variables should be screened before multiple linear regression analyses, therefore the sample size was predetermined as 240 (5 × 48).

### Measure instruments

The QLICP-BR (V2.0) was a scale of the QLICP system. It comprised 42 items with 32 items came from the general module QLICP-GM (V2.0) and 10 items from the module specific to breast cancer (SPD) ^23^. The QLICP-GM (V2.0) had the four domains: physical function (PHD), psychological function (PSD), social function (SOD), and common symptoms and side effects (SSD). Each item of the QLICP-BR (V2.0) was rated on the 5-point Likert scale of not at all, a little, a little, quite a bit, and very much. After reverse scoring of negatively worded items, the each domain score was obtained as the total of the corresponding item responses. The overall scale score was also obtained as the sum of the five domain scores. To ease comparison, all domain and total scale scores were standardized onto the 0–100 scale by subtracting its plausible minimum and then dividing the difference by the plausible range, in which a higher score indicates a better QOL.

The QLICP-BR (V2.0) has demonstrated good reliability, validity, responsiveness, and has been considered appropriate to comprehensively evaluate QOL of breast cancer patients in China^23^. According to this sample, its internal reliability was evaluated using the Cronbach’s alpha, which was 0.867 for the overall, and 0.626, 0.768, 0.626, 0.655, and 0.732 for the PHD, PSD, SOD, SSD and SPD, respectively. The item-domain correlation of a domain was generally greater in items belonging to the domain. Moreover, factor analysis coincides substantially with the theoretical conception. In addition, the responsiveness was also satisfactory with significant changes in the overall and all domain scores after treatments and the standardized response mean of 0.61 for the overall scale.

### Potential influencing factors

We collected socio-demographic data and disease-related characteristics, including sex, age, ethnicity, occupation, marital status, education, perceived income, medical insurance, drinking history, smoking history, treatment received, treatment effect, and treatment compliance (Table 1 in detail). We also collected 60 clinical or biochemical indexes from the hospital medical record information systems, including immunological tests such as specific protein detection and tumor markers tests, biochemical blood tests such as liver function tests, routine urine tests and tumor marker tests, blood routine, and so on. These factors could have potential influences on the QOL of patients with breast cancer.

**Table 1:** Socio-demographic and clinical characteristics of the sample (n = 246).

Characteristics	N	%	Characteristics	N	%
Ethnicity			Marital status		
Han	237	96.3	Married	239	97.2
Others	9	3.7	Others	7	2.8
Occupation			Age		
Worker	20	8.1	17–34	21	8.6
Farmer	112	45.5	35–54	143	58.1
Teacher	10	4.1	≥ 55	82	33.3
Officer/manager	13	5.3	Mean ($\overline{X} \pm S$)	50.07 ± 10.25	
Others	91	37.0	Education		
Perceived income			Primary school	65	26.4
Poor	52	21.1	Middle school	82	33.3
Fair	167	67.9	High school	66	26.8
High	27	11	Professional secondary school	27	11.1
Clinical stage			College	6	2.4
I	53	21.5	Medical insurance		
II	86	35	Self-paid	20	8.1
III	54	22	Urban and rural resident basic medical insurance	169	68.7
IV	27	11	Urban employee basic medical insurance	53	21.6
Missing	26	10.5	Commercial health insurance	4	1.6
Treatment received			Treatment effect		
Chemotherapy + radiation	3	1.2	Cured	25	10.2
Chemotherapy + surgery	39	15.9	Valid	52	21.1
Chemotherapy	113	45.9	Improved	155	63
Chemotherapy + radiation	3	1.2	Unchanged	6	2.4
Surgery	42	17.1	Deteriorated	2	0.8
Others	44	17.9	Others	6	2.4
Missing	2	0.8	Drinking history		
Treatment compliance			Yes	18	7.3
Absolutely not	13	5.3	No	228	92.7
A little bit	7	2.8	Smoking history		
Generally can	93	37.8	Yes	8	3.3
Most can	93	37.8	No	238	96.7
Absolutely can	40	16.3			

### Statistical analysis

All data analyses were conducted using SPSS 20.0. Descriptive statistics were used to summarize the socio-demographic variables and clinical parameters. Firstly, the influencing factors of each domain of PHD/PSD/SOD/SSD/SPD/TOT were analyzed by univariate analysis, respectively, considering too many factors. A simple linear correlation (Pearson correlation) was used for continuous variables, and t-test or one-way analysis of variance (ANOVA) was used for categorical variables. Secondly, considering too many variables in this survey (13 socio-demographic variables and 60 clinical or biochemical indexes), the statistically significant variables from the univariate analysis were fit into a multiple linear regression model to define independent influencing factors on QOL of PHD/PSD/SOD/SSD/SPD/TOT as dependent variables, respectively. The variables were selected by stepwise procedure with p-value in = 0.05 and p-value out = 0.10.

All methods were carried out in accordance with relevant guidelines and regulations.

### Ethics approval

The study involving human subjects were reviewed and approved by the IRB (Institutional Review Board) of Guangdong Medical University Hospital (PJ2012052, YJYS2019010). Subjects participated voluntarily and provided written informed consent.

### Consent to participate

All subjects gave written consent before they participated in the study.

## Results

### Characteristics of participants

A total of 246 participants were recruited. Table 1 summarizes their demographic and clinical characteristics. Their mean age was 50.07 years (SD = 10.25), with most of them were Han Chinese (96.3%), and the majority were farmers (45.5%). Most subjects had a moderate family economic status (67.9%) and were married (97.2%), and 8.1% of them did not have any health insurance.

### QOL in patients with breast cancer

Table 2 shows a summary of the QLICP-BR. The overall QOL in patients with breast cancer in China was 70.24 (SD = 8.70). The PHD scored 70.24 (SD = 11.24), PSD scored 69.48 (SD = 12.92), SOD scored 64.89 (SD = 10.43), SSD scored 83.96 (SD = 13.17), SPD scored 83.26 (SD = 12.16).

**Table 2:** QOL Scores Measuring by QLICP-BR (V2.0).

Domains	Mean	Standard deviation
Overall QOL	73.20	8.70
Physical function (PHD)	70.24	11.24
Psychological function (PSD)	69.48	12.92
Social function (SOD)	64.89	10.43
Common symptoms and side effects (SSD)	83.96	13.17
Specific module of the breast cancer (SPD)	83.26	12.16

### Factors influencing QOL

Table 3 lists the clinical and biochemical parameters that were significantly associated with the quality of life of breast cancer patients in univariate analyses.

**Table 3:** Values of selected clinical indicators that may affect the QOL in breast cancer.

Clinical objective indicators	N	Reference value	Means ± standard deviations	Minimum	Maximum
SP (mmHg)	243	90–140	119.52 ± 15.47	86.00	173.00
DP (mmHg)	243	60–90	76.28 ± 10.39	56.00	111.00
Pulse rate	243	66–100	84.70 ± 11.96	59.00	121.00
AST (U/L)	227	13–35	29.32 ± 32.01	11.00	282.00
ALP (U/L)	227	35–135	74.23 ± 50.67	30.00	486.00
GGT (U/L)	227	7–45	48.05 ± 93.84	6.00	895.00
Prototal protein (mg/L)	227	180–350	222.39 ± 57.19	30.00	422.90
FBG (mmol/L)	222	3.9–6.1	7.21 ± 32.49	3.53	489.00
Serum potassium ion level (mmol/L)	226	3.5–5.3	5.72 ± 25.48	3.02	387.00
Serum sodium ion level (mmol/L)	226	137–147	141.89 ± 2.54	129.00	147.60
Serum chloride ion level (mmol/L)	226	99–110	104.37 ± 2.73	91.90	115.00
Serum calcium ion level (mmol/L)	227	2.11–2.52	2.33 ± 0.12	1.91	2.70
Serum magnesium ion level (mmol/L)	224	0.75–1.02	0.85 ± 0.09	0.46	1.27
Serum phosphorus ion level (mmol/L)	226	0.85–1.51	1.23 ± 0.17	0.71	1.78
WBC (10^9^/L)	228	3.5–9.5	5.51 ± 2.01	2.57	20.78
Neut%	228	40–75	24.38 ± 36.57	0.29	344.80
Lymph%	229	20–50	12.11 ± 16.25	0.10	57.30
Mono%	229	3–10	2.27 ± 3.26	0.01	20.80
Eos%	229	0.4–8.0	0.65 ± 1.23	0.00	9.60
Baso%	229	0–1	0.20 ± 0.37	0.00	1.70
Neut# (10^9^/L)	229	1.8–6.3	3.54 ± 3.79	0.84	54.20
Lymph# (10^9^/L)	229	1.1–3.2	1.63 ± 0.61	0.41	3.73
BASO (10^9^/L)	229	0–0.06	0.02 ± 0.04	0.00	0.50
RBC (10^12^/L)	228	3.8–5.1	4.39 ± 2.21	3.10	36.90
Hb (g/L)	228	115–150	121.51 ± 15.95	13.90	161.00
Hct (L/L)	229	0.35–0.45	38.17 ± 5.27	3.87	62.30
MCV (fL)	229	82–100	91.11 ± 6.85	62.90	118.00
MCH (pg)	229	27–34	29.17 ± 4.99	18.50	94.30
MCHC (g/L)	229	316–354	315.97 ± 23.66	3.33	346.00
RDW (%)	228	11.5–14.5	27.71 ± 17.35	11.70	72.60
PLT (10^9^/L)	229	125–350	299.68 ± 92.83	75.00	703.00
PCT (%)	226	0.10–0.35	0.3 ± 0.08	0.08	0.73
MPV (fL)	226	9–13	10.43 ± 7.03	7.70	114.00
CEA (ng/mL)	206	≤ 5	12.06 ± 81.61	0.01	1124.38

*SP* systolic pressure, *DP* diastolic pressure, *AST* aspartate aminotransferase, *ALP* alkaline phosphatase, *GGT* γ-glutamyl transpeptidase, *FBG* fasting blood-glucose, *WBC* white blood cell, *Neut%* neutrophil ratio, *Lymph%* lymphocyte ratio, *Mono%* monocyte ratio, *Eos%* eosinophil ratio, *Baso%* basophil ratio, *Neut#* neutrophil count, *Lymph#* lymphocyte count, *BASO* basophil, *RBC* red blood cell, *Hb* hemoglobin, *Hct* hematocrit, *MCV* mean corpuscular volume, *MCH* mean corpuscular hemoglobin, *MCHC* mean corpuscular hemoglobin concentration, *RDW* red cell volume distribution width, *PLT* platelet, *PCT* platelet crit, *MPV* mean platelet volume, *CEA* carcinoembryonic antigen.

To identify the influential factors of HRQOL, multiple linear regression analyses were performed further to determine the effects of socio-demographic and selected clinical or biochemical indicators. Before multiple regression analyses, the categorical variables were recoded for quantification, as shown in Table 4. It will reduce levels of some variables such as Occupation (1 = Farmer, 0 = Others ), and also make the results clear and easy to understand. For example, the regression coefficient here means farmer higher than other occupation if it is positive, and vice versa.

**Table 4:** Quantification of categorical variables that may affect the QOL in breast cancer.

Factors	Quantifications
Occupation	1 = farmer,0 = others
Age	Actual value
Education	1 = primary school, 2 = middle school, 3 = high school, 4 = above high school
Perceived income	1 = poor, 2 = fair, 3 = high
Medical insurance	1 = completely self-paying, 0 = insured
Treatment received	1 = surgery, 0 = non-surgical
Treatment effect	1 = cured, 2 = valid, 3 = improved, 4 = unchanged, 5 = deteriorated
Treatment compliance	1 = absolutely not, 2 = a little bit, 3 = generally can, 4 = most can, 5 = absolutely can
Drinking history	1 = yes, 0 = no
Smoking history	1 = yes, 0 = no
Clinical biochemical indexes	Actual value

The results showed that either overall score or domains scores of QOL were influenced by some factors except for physical function (PHD). See Table 5 in detail.

**Table 5:** Predictors of domains and overall QOL selected by multiple linear regression.

Domains	Characteristics	Unstandardized coefficients		Standardized coefficients	T	P
		B	Std. error	Beta		
Overall QOL	Constant	7.675	31.039		0.247	0.805
	Medical insurance	−4.841	2.507	−0.130	−1.931	0.055
	Drinking history	−5.111	2.131	−0.164	−2.398	0.017
	ALP	−0.020	0.012	−0.125	−1.676	0.095
	Serum chloride ion level	0.454	0.242	0.140	1.875	0.062
	Serum calcium ion level	9.251	4.662	0.136	1.985	0.049
	Serum phosphorus ion level	−9.020	3.570	−0.173	−2.527	0.012
	MCV	0.282	0.100	0.219	2.815	0.005
	MCH	−0.223	0.125	−0.137	−1.782	0.076
	RDW	−0.101	0.037	−0.199	−2.734	0.007
	PLT	0.012	0.007	0.133	1.836	0.068
	Adjusted R square = 0.165, F = 4.885, P = 0.000					
Psychological function (PSD)	Constant	2.979	38.654		0.077	0.939
	Medical insurance	−7.312	3.617	−0.132	−2.022	0.045
	Drinking history	−7.734	3.092	−0.166	−2.501	0.013
	ALP	−0.041	0.017	−0.171	−2.389	0.018
	Serum chloride ion level	0.800	0.350	0.166	2.283	0.024
	Lymph%	−0.192	0.055	−0.229	−3.473	0.001
	PLT	0.016	0.009	0.115	1.751	0.081
	Adjusted R square = 0.201, F = 9.241, P = 0.000					
Social function (SOD)	Constant	65.073	14.466		4.499	0.000
	Medical insurance	−5.112	2.567	−0.131	−1.991	0.048
	Drinking history	−6.546	2.607	−0.163	−2.511	0.013
	Serum potassium ion level	0.056	0.026	0.138	2.172	0.031
	Serum calcium ion level	9.517	5.704	0.111	1.668	0.097
	Lymph%	−0.196	0.049	−0.297	−4.030	0.000
	MCH	−0.256	0.135	−0.123	−1.899	0.059
	Treatment compliance					
	Absolutely not	−10.732	4.289	−0.168	−2.502	0.013
	A little bit	−4.464	4.044	−0.075	−1.104	0.271
	Generally can	1.755	2.152	0.080	0.816	0.416
	Most can	0.915	2.045	0.042	0.447	0.655
	Absolutely can			Reference		
	Adjusted R square = 0.175, F = 5.482, P = 0.000					
Common symptoms and side effects (SSD)	Constant	80.461	1.990		40.429	0.000
	CEA	−0.022	0.010	−0.145	−2.102	0.037
	Treatment compliance					
	Absolutely not	−0.547	5.774	V0.007	−0.095	0.925
	A little bit	5.313	4.989	0.079	1.065	0.288
	Generally can	7.336	2.444	0.286	3.002	0.003
	Most can	3.353	2.408	0.133	1.393	0.165
	Absolutely can			Reference		
	Adjusted R square = 0.129, F = 2.833, P = 0.000					
Specific module of the breast cancer (SPD)	Constant	92.167	8.532		10.803	0.000
	Age	0.241	0.079	0.209	3.041	0.003
	Serum phosphorus ion level	−11.353	5.222	−0.157	−2.174	0.031
	RDW	−0.130	0.058	−0.185	−2.215	0.028
	CEA	−0.022	0.010	−0.154	−2.247	0.026
	Perceived income			Reference		
	Poor	−5.302	2.094	−0.180	−2.532	0.012
	Fair					
	High	1.476	2.561	0.042	0.576	0.565
	Treatment effect			Reference		
	Cured	0.948	3.698	0.023	0.256	0.798
	Valid	−5.942	2.248	−0.204	−2.643	0.009
	Improved			Reference		
	Unchanged	1.818	5.528	0.024	0.329	0.743
	Deteriorated	−16.294	11.284	−0.138	−1.444	0.150
	Others	−6.776	7.014	−0.098	−0.966	0.335
	Treatment compliance					
	Absolutely not	−10.995	6.029	−0.146	−1.824	0.070
	A little bit	7.256	7.862	0.114	0.923	0.357
	Generally can	−0.035	2.756	−0.001	−0.013	0.990
	Most can	−0.257	2.494	−0.011	−0.103	0.918
	Absolutely can			Reference		
	Adjusted R square = 0.129, F = 2.931, P = 0.000					

For overall QOL, the multiple linear regression analyses indicated that the factors having significant associations with the QOL in patients with breast cancer were medical insurance, drinking history, alkaline phosphatase (ALP), serum chloride ion, serum calcium ion, serum phosphorus ion, mean corpuscular volume (MCV), mean corpuscular hemoglobin (MCH), red cell volume distribution width (RDW) and platelet (PLT). The model explained 16.5% of the variability of QOL (Adjusted R Square = 0.165).

For the individual dimensions, there were no significant variables associated with PHD, and there was one more significant variable associated with other domains. The results showed that factors having significant associations with PSD were medical insurance, drinking history, ALP, serum chloride ion level, lymphocyte ratio (Lymph%), and PLT, which explained 20.1% of the variability of QOL (Adjusted R Square = 0.201). The factors which most influenced QOL in social function were medical insurance, drinking history, serum potassium ion level, serum calcium ion level, Lymph%, MCH, and treatment compliance, which explained 17.5% of the variability of QOL (Adjusted R Square = 0.175). In regard to SSD, the results indicated that the influencing factors of QOL included carcinoembryonic antigen (CEA) and treatment compliance, which explained 12.9% of the variability of QOL (Adjusted R Square = 0.129). For SPD, the results indicated that the influencing factors of QOL included age, serum phosphorus ion level, RDW, CEA, perceived income, treatment effect, and treatment compliance, which explained 12.9% of the variability of QOL (Adjusted R Square = 0.129).

## Discussions

This is the first study that employed the QLICP-BR (V2.0) scale for assessing QOL in patients with breast cancer. It revealed the current status of QOL and its influencing factors for breast cancer patients in China. The results served as a reference for further research on the QOL of breast cancer patients.

Our findings emphasized that the general QOL for our study population was fairly good. At domains level, SSD scored the highest, SOD scored the lowest. The social functioning reflects the ability and satisfaction of patients to participate in social roles and activities in life and work. The low score of this domain may be because only 13.5% of the subjects in this study had higher education and most of them (45.5%) were farmers with low economic and social status, which was consistent with the findings of Coughlin et al^24^. The results of the study by Coughlin et al.^24^ showed that low-income and poorly educated breast cancer patients have relatively limited access to social and medical resources and need to consider both disease and treatment for their families. These factors may affect the interest and frequency of patients' participation in previous social roles and activities, and their access to social resources is relatively limited. Therefore, their ability and satisfaction with social roles and activities were low.

Many researchers^25–28^ have found that the scores and decrements in scores of HRQOL in patients with breast cancer were associated with socioeconomic and demographic factors such as economic status, educational attainment, and age. Our results also confirmed some of these factors, with HRQOL in breast cancer were influenced by medical insurance, drinking history, age, etc. The International Agency for Research on Cancer^26^ has concluded a causal relationship between alcohol and the risk of breast cancer. Alcohol consumption is a risk factor for breast cancer, but the influence of alcohol consumption on QOL is less well known^27^. However, alcohol may play a role in the initiation and promotion of breast tumors by increasing sex hormone levels, enhancing the responsiveness of breast epithelial cells to sex hormones, and producing genotoxic metabolites acetaldehyde and oxidative stress^28^. And there is evidence^29^ that alcohol promotes the progression and prognosis of breast tumors. Therefore, linking alcohol consumption to the QOL of breast cancer patients can also provide a basis for advising breast cancer patients to prohibit or reduce alcohol consumption to some extent.

The form of medical insurance also impacts the QOL of breast cancer patients, and the QOL of insured breast cancer patients was significantly better than that of patients who were completely self-paying. The same was true for the total QLICP-BR (V2.0) score as well as the psychological function and social function scores. This study is similar to the study of QOL determinants in breast cancer patients carried out in Shanghai, China^30^. This finding suggests that providing financial support to breast cancer patients through government financial subsidies and other support mechanisms may considerably improve the QOL of breast cancer patients.

In addition to socio-demographic factors, clinical indicators which influenced QOL in these patients were those related to disease severity, such as ALP, Ca^2+^, Cl^−^, P^3−^, MCV, MCH, RDW, and PLT, etc. This was true for QLICP-BR (V2.0) total quality of life, and five functioning scores each.

The study^31^ has shown that ALP is the independent risk factor for bone metastases in patients with breast cancer. Breast cancer patients with bone metastases have significantly lower five-year survival rates and can present serious complications, which reduces the QOL of the patients^32^. This study had confirmed the tendency. Therefore, early detection and diagnosis of bone metastases in breast cancer patients can be helpful for treatment.

Serum calcium ion level and serum chloride ion level had a positive effect on the QOL score, and serum phosphorus ion level had a negative effect on the QOL score. However, the roles of ion channels and acid–base disturbances in tumorigenesis remain unclear^33^. In addition, the expression and activity of certain membrane channels have been associated with cancer progression, their effects on oncogenic signaling pathways remain largely elusive^34^. Therefore, the current study^35–38^ is focusing on exploring the possible role of intracellular ion channels in cancer development and progression and on developing new drugs that can modulate the expression and/or activity of these channels on the other hand. Because no study has confirmed that Ca^2+^, Cl^−^ and P^3−^ are related to the quality of life, whether they can affect the QOL of breast cancer patients remains to be further studied.

However, the association between RBC parameters and the development of venous thromboembolism appears to be weak in cancer patients and independent of other identified risk factors (e.g., cancer transmission)^39^. Therefore, the impact of MCV, MCH, and RDW on the QOL in breast cancer needs to be further explored. Furthermore, platelet had a positive effect on the QOL score in this study. Considerable evidence has indicated that the roles of platelet in the progression of breast cancer, including increased survival of disseminated cancer cells within the circulation, tumor cell adhesion to endothelial cells, parenchymal infiltration of distant tissues, and ultimately tumor cell growth at metastatic sites, which does not accord with the results of our research, awaits further research ^40–46^.

For the individual dimensions, clinical indicators were strong factors affecting QOL. The reason may be that the objective clinical indexes reflect the severity of the disease at that time, and the clinically relevant objective indexes are poor during the acute phase, which also seriously affects the psychological, social, and physiological functions of patients, thus leading to poor QOL of patients at that time. But, the relationship between clinical biochemical indexes and QOL still needs to be further studied.

In conclusion, we investigated factors affecting the QOL of breast cancer patients, results showed that the influential factors of breast cancer patients’ QOL were affected by many factors. Targeted management or intervention strategies should be developed based on the research of the impact on the QOL of breast cancer patients, aiming to improve their QOL.

There are some limitations to this study. First, the patients were sampled only in Guangdong and Yunnan provinces, and the sample may not be representative of the general population of Chinese breast cancer patients, but the results of this study can be compared with other similar studies. Second, there may be other potential factors that affect the QOL of breast cancer patients, which were not comprehensively explored in this study. Besides, only statistically significant variables in the single factor analysis were included in the multiple linear regression model, considering there were too many independent variables relative to the sample size and the selection of too many variables would have resulted in a decrease in precision due to the excessive amount of calculations. And thus it could have resulted in omission of some variables with a large interaction and a small individual effect. Last but not least, this study focused on an initial exploration of the demographic and clinical characteristics of factors that may influence the QOL of breast cancer patients, the mechanisms of QOL influencing factors remain unclear especially clinical biochemical indexes, which still need to be further studied.

## Data Availability

The data used to support the findings of this study are available from the corresponding author upon request.
